# Differences in sickness absence between self-employed and employed doctors: a cross-sectional study on national sample of Norwegian doctors in 2010

**DOI:** 10.1186/1472-6963-14-199

**Published:** 2014-05-02

**Authors:** Judith Rosta, Gunnar Tellnes, Olaf G Aasland

**Affiliations:** 1 Institute for Studies of the Medical Profession, Oslo, Norway; 2 Institute of Health and Society, University of Oslo, Oslo, Norway

**Keywords:** Sickness absence, Doctors, Work stress, Norway

## Abstract

**Background:**

Doctors have a low prevalence of sickness absence. Employment status is a determinant in the multifactorial background of sickness absence. The effect of doctors’ employment status on sickness absence is unexplored. The study compares the number of sickness absence days during the last 12 months and the impact of employment status, psychosocial work stress, self-rated health and demographics on sickness absence between self-employed practitioners and employed hospital doctors in Norway.

**Methods:**

The study population consisted of a representative sample of 521 employed interns and consultants and 313 self-employed GPs and private practice specialists in Norway, who received postal questionnaires in 2010. The questionnaires contained items on sickness absence days during the last 12 months, employment status, demographics, self-rated health, professional autonomy and psychosocial work stress.

**Results:**

84% (95% CI 80 to 88%) of self-employed and 60% (95% CI 55 to 64%) of employed doctors reported no absence at all last year. In three multivariate logistic regression models with sickness absence as response variable, employment category was a highly significant predictor for absence vs. no absence, 1 to 3 days of absence vs. no absence and 4 to 99 days of absence vs. no absence), while in a model with 100 or more days of absence vs. no absence, there was no difference between employment categories, suggesting that serious chronic disease or injury is less dependent on employment category. Average or poor self-rated health and low professional autonomy, were also significant predictors of sickness absence, while psychosocial work stress, age and gender were not.

**Conclusion:**

Self-employed GPs and private practice specialist reported lower sickness absence than employed hospital doctors. Differences in sickness compensation, and organisational and individual factors may to a certain extent explain this finding.

## Background

It is documented that the sickness absence in the workforce sectors with higher rate of self-employment is lower than in the public sector [1]. In Norway, hospital doctors are salaried employees with no salary reduction during sickness absence, whereas private practice specialists and general practitioners (GPs) as self-employed may contract economic losses during their eventual sickness absence, with 16 waiting days and a compensation of 40% of gross profit, with an annual maximum of NOK 711,900 in 2010 (EUR 93,680; GBP 76,020) [2,3]. An additional difference between the two groups exists in the commitment for continuous patient care. If absent from work, private practitioners must usually find a locum as opposed to employed hospital doctors [2]. This can be a challenge, as most GPs and private practice specialists work alone or in small groups, with an increasing demand for GPs and private practice specialists in Norway [4].

Work stressors are important determinants in the multifactorial background of sickness absence [5-8]. For doctors, a high level of work stress combined with low prevalence of sickness absence is a familiar situation [5,7,9-15]. Some studies suggest an association between poor team work, bullying and sickness absence among different categories of hospital staff [7,16].

A Norwegian study with data from 1993 showed that working whilst ill was more common among GPs compared with hospital doctors [17]. A preliminary analysis of the 2010 postal survey among Norwegian doctors suggests that the majority of doctors have little or no sickness absence at all – particularly doctors in general and specialist practice [9].

We have previously shown that in Norway every fourth GP compared to every third senior hospital doctor perceive their workload as unacceptable, and that GPs and other private practitioners through repeated surveys from 1994 have been more satisfied in their jobs than have hospital doctors [9,18,19] – suggesting less job stress [20]. Among employees in general an inverse relationship between job satisfaction and sickness absence has been documented [6].

Studies on work and stress are often based on one of two stress models. Karasek’s job strain model is widely used to describe how decision latitude (professional autonomy), psychological demands and social support, may have a bearing on sickness and sickness absence [21]. Decision latitude is negatively associated with sickness absence, while the effects of psychological demands and social support at work are not so clear [22]. Another stress model, the effort-reward imbalance model (ERI) has been proposed by Siegrist [23]. According to this model, a lack of balance between effort and reward leads to emotional distress and increases the risk of poor health and sickness absence [15,24-26].

In this study we apply the ERI-model, supplemented by a measurement of the level of decision latitude.

The aim of this study is to compare the number of sickness absence days during the last 12 months and the impact of the employment status, psychosocial work stress, self-rated health and demographics on sickness absence between self-employed practitioners and employed hospital doctors in Norway.

## Methods

### Data collection and sample

In Norway, doctors’ health and working conditions have been studied in an extensive research program by The Research Institute of the Norwegian Medical Association starting in 1992. Since 1994, a representative panel of originally 1,200 active Norwegian doctors has been followed through postal questionnaires biennially. Over the years, younger doctors have been included and several hundred have left the panel due to retirement, death or voluntary withdrawal. The present study is based on data from 2010, with 1,014 respondents out of 1,520 potential.

### Ethics

The project is in compliance with the declaration of Helsinki on Ethical Principles for Medical Research Involving Human Subjects adopted by the World Medical Association (http://www.wma.net/en/30publications/10policies/b3/index.html). According to the Regional Committee for Medical Research Ethics, the study based on “Norwegian Physician Survey - A bi-annual prospective questionnaire survey to a representative sample of Norwegian physicians” is exempt from review in Norway, cf. §§ 4 of The Act. The project can be implemented without the approval by the Regional Committee for Medical Research Ethics (IRB 0000 1870). Additionally, approval for data protection of the bi-annual prospective survey among Norwegian doctors was obtained from the Norwegian Social Science Data Service (Reference 19521).

### Measurements

#### Sickness absence

Sickness absence was measured with a single question: “How many days of sickness absence have you taken during the past 12 months?” No distinction was made between doctor-certified and self-declared absence, or whether the sickness absence days were taken as one or several spells during the year. Preliminary analyses of the data showed [9] that the majority of doctors had none or only a few days of sickness absence during the last 12 months. Therefore, the reported numbers of sickness absence days were split into four levels: 0 days, 1–3 days, 4–99 days, and 100 or more days. Sickness absence days and sick days were used as synonyms in the study.

#### Potential determinants of sickness absence days

*Psychosocial stress at work* was measured by the validated short form of the effort-reward questionnaire (ERI) [27]. It comprises four items from the effort scale (time pressure, interruptions/disturbances, responsibility and demanding in the job) and five items from the reward scale (remuneration, esteem/appreciation, career opportunities such promotion, job security). Estimations were given on a five point Likert scale. According to this model, work stress is rooted in a chronic mismatch between high efforts and low rewards. Hence, a ratio of the sum score of the effort items (nominator) relative to sum score of the reward items (adjusted for the number of items; denominator) greater than one indicates a high level of psychosocial work stress.

*Professional autonomy* (decision latitude) was measured with one item from the validated job satisfaction scale of Warr et al. [28] “How satisfied are you with the freedom to choose your own methods of working?”, scored on a seven-point scale from very dissatisfied to very satisfied.

*Weekly working hours* were recorded as a self-estimated continuous variable [19].

*Self-rated health,* measured by the question “In general, would you say your health is: very good, good, not very good (average), poor”. This item has been showed to be correlated with mortality risk, morbidity, and general health status [29].

Other variables were *sex* and *age,* plus the main grouping variable dividing the sample in two: *employed hospital doctors* (consultants and interns) and *self-employed doctors* (general practitioners and private practice specialists).

### Analyses

We used Pearson’s Chi-square test for categorical data, and ANOVA for comparison of group means. Logistic regressions were used to estimate the simultaneous effect of gender, age, self-rated health, employment status, professional autonomy and work stress caused by psychosocial work environment on sickness absence during the last 12 months. SPSS Statistics, version 19 was used for the analyses.

## Results

### Sample characteristics

The response rate was 67% (1,014/1,520). 948 doctors younger than 68 years answered the question on number of sickness absence days. 521 of these worked as employed consultants or interns in hospital trusts and 313 as self-employed GPs and private practice specialists. 103 worked in other settings (administration, research), and they are not included in the subsequent analyses. 11 respondents who did not indicate their present work situation were also excluded.

The proportions of GPs (30.5%; 254/834), private practice specialists (7%; 59/834) and consultants and interns in hospital trust (62.5%; 521/834) were not significantly different from the same segments of the total Norwegians doctor workforce (consultants and interns 65%, 10,907/16,793; GPs 29.4%, 4,942/16,793; private practice specialists 5.6%, 944/16,793). Gender and age proportions were also roughly the same (data not shown).

### Potential determinants of sickness absence

Table 1 presents the potential determinants of sickness absence among doctors by employment status. Males, older age, high professional autonomy and low psychosocial work stress were more prevalent among self-employed than employed doctors. There were no differences in weekly working hours or self-rated health.

**Table 1:** Potential determinants of sickness absence of Norwegian doctors by employment status in 2010

	**Employed doctors**	**Self-employed doctors**	**p-value**
	**(n = 521)**	**(n = 313)**	
**Gender** (%)			
Female	43.0	32.8	
Male	57.0	67.2	0.003
[Missing, n]	[n = 0]	[n = 0]	
**Age**, year (%)			
29-35	13.9	6.5	
36-44	31.4	20.1	
45-67	54.7	73.4	0.0001
[Missing, n]	[n = 0]	[n = 0]	
**Working hours/week **(%)			
≤40	30.3	32.8	
41-48	41.5	39.7	
>48	28.2	27.5	0.641
[Missing, n]	[n = 18]	[n = 13]	
**Self-rated health** (%)			
Very good	41.4	39.5	
Good	45.9	46.7	
Average	11.9	13.1	
Poor	0.8	0.7	0.929
[Missing, n]	[n = 7]	[n = 17]	
**Professional autonomy** (mean)			
(From 1 very dissatisfied to 7 very satisfied)	4.9	5.7	0.0001
[Missing, n]	[n = 12]	[n = 12]	
**Levels of psychosocial work stress** (%)			
High levels (ERI ration >1)	24.3	11.4	
Low levels (ERI ratio ≤1)	75.6	88.6	0.0001
[Missing, n]	[n = 32]	[24]	

Comparisons of proportions by Pearson’s Chi-square test and of interval variables by ANOVA.

### Number of sick days

**Figure 1:**
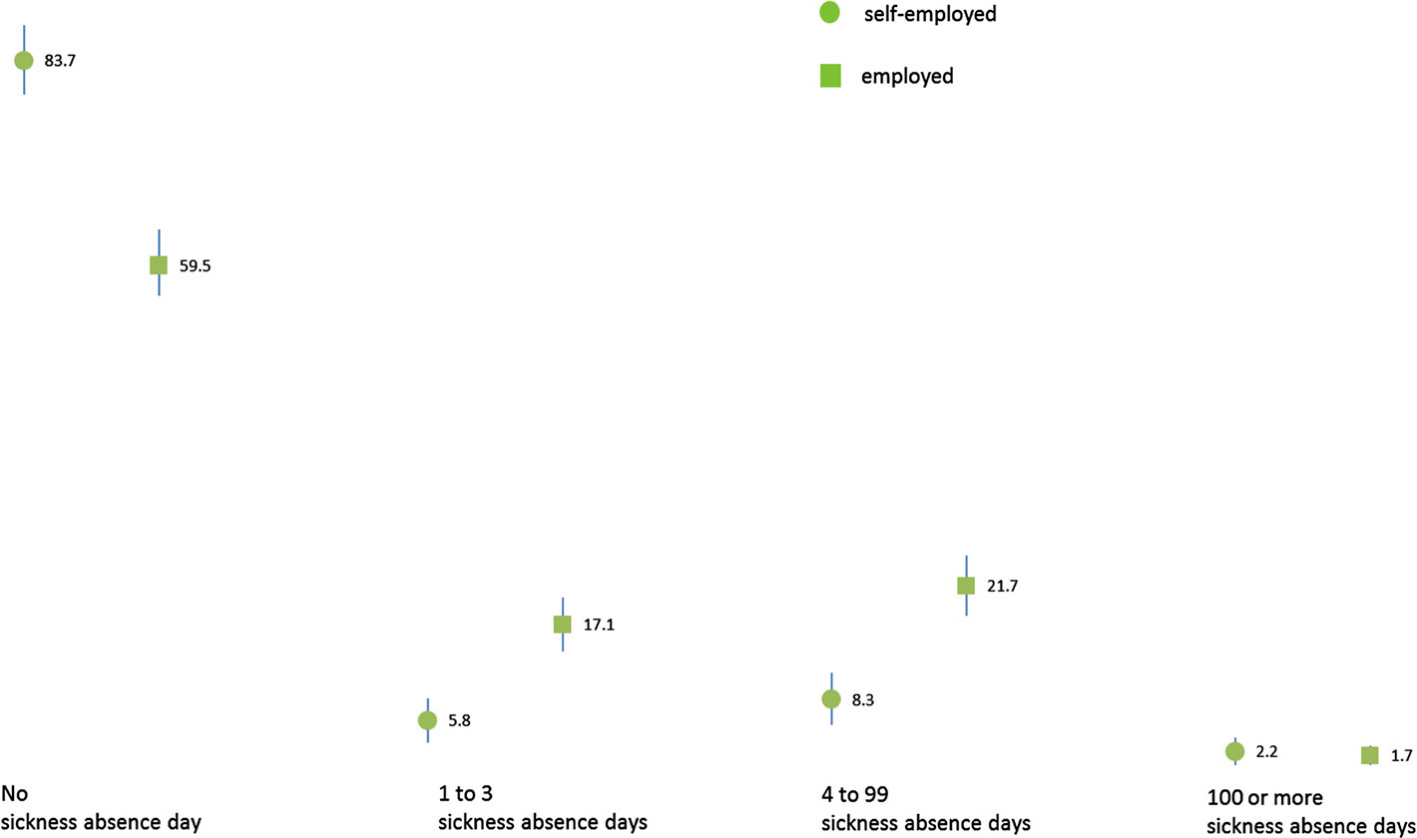
Levels of the sickness absence days during the last 12 months among Norwegian doctors by employment status in 2010.

Figure 1 illustrates the levels of the sickness absence days during the last 12 months among Norwegian doctors by employment status in 2010. 83.7% (95% CI 80 to 88%) of self-employed doctors and 59.5 (55 to 64)% of employed doctors reported no sickness absence at all last year. 5.8 (3 to 8)% of the self-employed and 17.1 (14 to 20)% of the employed doctors reported 1 to 3 sickness absence days, and 8.3 (5 to 11)% of the self-employed and 21.7 (18 to 25)% of the employed doctors reported 4 to 99 sickness absence days. Judged by the confidence intervals, the differences between self-employed and employed doctors were statistically significant on all three levels. With regard to the small group with a very high number of sickness absence days, there was no difference, 2.2 (1 to 4)% (7/313) of the self-employed and 1.7 (1 to 3)% (9/521) of the employed doctors reported 100 days or more.

### The importance of employment category

To further explore the differences between the two categories of doctors in this study we performed a multivariate logistic regression with doctor category as the response variable (Table 2). The employed doctors were younger, had better self-rated health, were slightly more likely to work more than 40 hours per week (not significant), experienced a significantly lower professional autonomy and more psychosocial work stress. The statistically significant differences in sick days remained when controlled for the above mentioned variables. There was no gender effect in this model.

**Table 2:** Logistic regression model on employment status as response variable among Norwegian doctors in 2010 (method: enter; n = 768)

	**Employment status of Norwegian doctors**		
	**(1 = employed; 0 = self-employed)**		
	**OR**	**95% CI**	**P**
**Age in years**	0.98	0.96-0.99	0.005
**Male** (vs. female)	0.76	0.53-1.08	0.120
**Self-rated health as very good or good** (vs. average/poor)	2.09	1.18-3.40	0.010
**>40 working hours/week** (vs. **≤**40 hours)	1.40	0.97-2.00	0.069
**Professional autonomy** (score 1–7)	0.60	0.51-0.70	0.0001
**High levels of psychosocial work stress** (vs. low levels)	1.61	1.01-2.56	0.045
**Sickness absence days/12 months**			
Non (reference)	1		
1-3	3.78	2.14-6.67	0.0001
4-99	3.67	2.13-6.30	0.0001
100 or more	1.24	0.35-4.42	0.744

### Variations in number of sick days

We also looked at differences related to number of sick days. Table 3 shows four multivariate logistic regression models with different levels of sick days as response variables. The predictors are employment category, age, gender, self-rated health, weekly working hours, professional autonomy and perceived psychosocial work stress (ERI).

**Table 3:** Logistic regression models on sickness absence days during the last 12 months as response variable among Norwegian doctors in 2010 (method: enter)

	**Multivariate analysis**											
	**Model 1**			**Model 2**			**Model 3**			**Model 4**		
	**≥1 sick days (n = 236)**			**1-3 sick days (n = 100)**			**4-99 sick days (n = 122)**			**100 or more sick days (n = 14)**		
	**(vs. none sick days, n = 532)**			**(vs. none sick days, n = 532)**			**(vs. none sick days, n = 532)**			**(vs. none sick days, n = 532)**		
	**OR**	**95% CI**	**P**	**OR**	**95% CI**	**P**	**OR**	**95% CI**	**P**	**OR**	**95% CI**	**P**
**Age**	1.01	0.99-1.02	0.529	1.00	0.98-1.02	0.994	1.00	0.98-1.03	0.790	1.07	1.00-1.14	0.048
**Male** (vs. female)	0.95	0.66-1.36	0.777	1.24	0.76-2.01	0.387	0.81	0.51-1.28	0.369	0.43	0.12-1.64	0.218
**Self-rated health as very good/good** (vs. average/poor)	0.29	0.18-0.47	0.0001	0.71	0.34-1.50	0.371	0.20	0.12-0.35	0.0001	0.09	0.03-0.30	0.0001
**>40 work hours/week** (vs. **≤**40 hours)	0.51	0.35-0.72	0.0001	0.51	0.32-0.81	0.004	0.57	0.36-0.89	0.014	0.34	0.10-1.18	0.090
**Professional autonomy** (score 1–7)	0.82	0.71-0.94	0.005	0.87	0.72-1.06	0.157	0.82	0.69-0.97	0.023	0.56	0.35-0.88	0.012
**High levels of psychosocial work stress** (vs. low levels)	0.96	0.63-1.47	0.854	0.95	0.53-1.70	0.871	0.99	0.58-1.69	0.968	0.46	0.09-2.35	0.349
**Employed doctors** (vs. self-employed doctors)	3.34	2.24-4.98	0.0001	3.74	2.13-6.56	0.0001	3.32	1.95-5.66	0.0001	0.83	0.23-2.93	0.766

Age, gender and perceived psychosocial work stress were not significant predictors in any of the models. Employment category was highly significant except for those with more than 100 sick days last year (model 4), indicating that serious chronic disease or injury is not related to employment category (see also Table 2). Self-rated health and professional autonomy are significant predictors of sickness absences of more than 3 days.

## Discussion

### Principal findings

Employed hospital doctors reported higher sickness absence compared with self-employed GPs and private practice specialists. Age, gender and psychosocial work stress measured by ERI had no impact on the number of sick days.

### Comparisons with other studies

A comparison with other studies is limited by methodological differences regarding data, sample characteristics and measurements methods. However, Norwegian doctors seem to have a poor effort-reward balance as 19% (95% CI 13.3-25.6) had an ERI ratio greater than one, compared with 5.6% (95% CI 1.0-10.2) of high-skilled Norwegian white collar workers [30]. With regard to sick days, the number of yearly working days lost per full-time equivalent Norwegian employee according to the European Labour Force Survey in 2004 was 22, compared with 7 days for doctors [31]. Neither is the higher risk of sickness absence among females in the Norwegian workforce found among the doctors [32].

The international literature is ambiguous on the issues of doctors’ sickness absence and work stress. While we find that employed hospital doctors run a higher risk of sickness absence and psychosocial work stress than self-employed doctors in private practice, a study from the UK reports no difference between GPs and other doctors in sick leave [12]. In another UK study, higher percentage of GPs than hospital doctors perceived their job as “often or always stressful” (69% vs. 51%), and reported no sick leave in the last year (females: 65.2% vs. 42.9%; males 65% vs. 58%). However, more occupational stress and fewer days sick leave were reported by GPs and hospital doctors than company fee earners [13]. In Germany, psychosocial work stress as measured by ERI, was similar among hospital doctors in surgical fields (female: 24%; male: 26%) and doctors in private practice (28%) [15,20]. A Finnish study found only a small difference between GPs and consultants in psychological stress using the 12-item version of the General Health Questionnaire, and that Finnish GPs had more short-term, but not long term, sickness absence than consultants [33]. To report no sick days at all was more frequent among our Norwegian doctors than in a comparable UK study [12].

These international differences in doctors’ sick days and stress levels may be due to different measurement instruments and different types of contract or sickness compensation schemes. In Norway, 94% of the GPs, as opposed to their hospital employed colleagues, have limited sickness compensation and obligatory waiting days, while 92% of Finnish GPs are employed by municipal health centres and have the same sickness compensation scheme as hospital doctors [2,34]. In the UK, all NHS doctors, most hospital doctors and about a third of the GPs have a sickness compensation scheme. For the self-employed GPs sickness compensation arrangements depend on locum insurance [34-36].

Different from studies in the general population and professional groups, the high ERI levels of psychosocial work stress was not associated with number of sick days in our sample [22-26]. On the other hand, and consistent with other research using Karasek’s job stain model, professional autonomy was a significant negative predictor of sickness absence of more than three days [22]. In line with other studies, self-rated health was also a significant negative predictor of sickness absence in our sample [6].

### Explanation of the results

It is clear that the type of sickness compensation has a bearing on the amount of sickness absence [22]. In Norway the introduction of full compensation for wage earners from day one in 1978 led to increased sickness absence [37]. Norwegian doctors employed by health trusts (mainly hospitals) are entitled to this type of sickness compensation, while the self-employed doctors (general practitioners and private practice specialists) usually have to wait at least 16 days, and do not get full compensation. In addition, these doctors are likely to be more committed to continuous patient care and must find a locum in their absence [2].

The potential effect of attitude toward sickness absence must be also considered [22]. We have data on sick days from 442 hospital doctors who answered the survey both in 2008 and 2010. 70% (17/20) of the doctors who had become self-employed reported no sick days in 2010, compared to 53% (224/422) of those who were still working in hospitals, a statistically significant difference (p = 0.005, Pearson’s Chi-square Test).

Also among the 13% of all doctors who rated their own health to be average or poor the self-employed more often reported no sick days last year (57% vs. 43%, p = 0.032; Pearson’s Chi-square Test). This is in line with an investigation using Norwegian data from 1993 showing higher prevalence of working whilst ill among self-employed GPs than employed hospital doctors [17].

It is possible that the observed difference in sickness absence between the two groups may be associated with the nature of the job rather than employment status. A Finnish study describes the working environment for doctors in the public sector as more strenuous than for doctors in the private sector, while a UK study reports more work stress among GPs than among hospital doctors [13,38]. Other studies show no or small differences in perceived psychological stress between GPs and hospital doctors [15,20,33]. In our sample, hospital doctors compared with GPs and private practice specialists experienced more psychosocial work stress (Table 2). However, we found no association between psychosocial work stress and number of sick days (Table 3). Our studies with data from the last decade suggest similarities in the nature of job of employment and self-employment doctors in Norway. Several lifestyle factors such as alcohol consumption, smoking and physical activity were similar across doctor groups [9]. We also showed that the perceived unacceptable work stress, working hours and a desire for a change in workload did not differ significantly between hospital and private practice doctors [9,19].

### Policy implications

A good professional climate and reduction of psychosocial risks at the workplace are reflected in European and the Norwegian working conditions legislature [39,40]. Doctors, particularly those working in hospitals, show an increased risk for psychosocial work stress. High doctor work stress is related negatively to quality of health care and positively to a number of physical and mental disorders such as burnout [15]. A current Norwegian study shows that sickness absence after a counselling intervention for burnout can prevent later burnout [41]. Almost 70% of the doctors in our sample reported no sick days during last year, but 13% rated their own health as average or poor. We know that doctors go to work with the same symptoms that they certify sick their patients for, which may negatively affect both their health and job satisfaction [6,17]. Therefore, a lower threshold for sickness absence is required. Good doctor health is a necessary prerequisite for a good quality and stability of our total health care systems.

### Limitations and strengths

The strength of this study lies first and foremost in the representative dataset, making the results generalizable to the entire population of doctors in hospital and private practice in Norway. The high validity of the instruments concerning on Effort-Reward Imbalance, professional autonomy and the self-rated health are also strengths of the study [27-29].

The response rate of 67% is fairly good. It is higher than in a number of other doctor studies, but does not rule out the possibility of non-respondents bias. There is of course the possibility that the doctors who did not respond had a long-term absence from job due to illness.

A further limitation is that self-reported sickness absence days that cannot be controlled against official records. However, there is at least one study that shows agreement between self-reported and recorded number of sickness absence days over a 12 month period [42].

The duration and type of sickness absence episodes (self-declared or doctor-certified) were not recorded in this survey, neither were the reasons for sickness absence, nor whether the reasons were work related. However, it is well documented that a significant part of the sickness absence are work-related [22]. Female doctors are more involved with family care than their male counterparts, and some of the reported sick days could be related to childcare or care of other family members [43]. A recent Norwegian study shows that sickness absence among females are mostly related to caring responsibilities, while the men have more stress and conflicts at work [44].

In our study, private practice specialists and GPs were defined as self-employed doctors. However, there is a small group of GPs (5.7% in 2010) in Norway who by choice are employed by the municipalities with a fixed salary and a sickness compensation system similar to the hospital doctors [4]. We are not able to identify these doctors in our sample.

One might speculate whether the different proportions of GPs (254/313) and specialists in private practice (54/313) in the self-employment group, and senior (405/521) and junior (116/521) doctors in the employment group may affect the results, but this does not seem to be the case. Among hospital doctors the level of sick days was similar between juniors and seniors, as was the level between general practitioners and other private practice specialists (data not shown).

Because sickness absence varies between cultures and the number of foreign doctors in Norway is increasing, it is also important to include this perspective in further research on doctors’ sickness absence [31,45]. Other specific elements in doctors’ work situation like concerns about clinical responsibility, emotional burden of patient care or finding a locum on short notice might be also useful, which was not possible in the present study [1,17,22].

## Conclusion

The study emphasises the role of employment category in sickness absence among Norwegian doctors. Self-employed GPs and private practice specialists report less sickness absence than employed interns and consultants in the hospital. Differences in sickness compensation, and organisational and individual factors may to a certain extent explain this finding.

## Competing interests

None of the authors have any conflict of interests to declare.

## Authors’ contributions

JR and OGA contributed to concept and design of the study, analysis and interpretation of the data, and writing the article. GT contributed to concept of the study and critical revision of the manuscript. All authors read and approved the final manuscript.

## Pre-publication history

The pre-publication history for this paper can be accessed here:

http://www.biomedcentral.com/1472-6963/14/199/prepub
